# Laparoscopic versus hybrid approach for treatment of incisional ventral hernia: a 5–10-year follow-up of the randomized controlled multicenter study

**DOI:** 10.1007/s10029-023-02849-1

**Published:** 2023-08-18

**Authors:** J. M. Hiekkaranta, M. Ahonen, E. Mäkäräinen, J. Saarnio, T. Pinta, J. Vironen, S. Niemeläinen, P. Vento, M. Nikki, P. Ohtonen, T. Rautio

**Affiliations:** 1 Department of Surgery, Oulu University Hospital, Oulu, Finland; 2 Department of Radiology, Oulu University Hospital, Oulu, Finland; 3 Department of Surgery, Seinäjoki Central Hospital, Seinäjoki, Finland; 4 Department of Surgery, Helsinki University Hospital, Helsinki, Finland; 5 Department of Surgery, Tampere University Hospital, Tampere, Finland; 6 Department of Surgery, Kymenlaakso Central Hospital, Kotka, Finland; 7 Research Service Unit, The Research Unit of Surgery, Anesthesia and Intensive Care, Oulu University Hospital, University of Oulu, Oulu, Finland

**Keywords:** Incisional hernia, Incisional ventral hernia, Laparoscopy, Hybrid, Recurrence, Quality of life, Chronic pain

## Abstract

**Purpose:**

In this long-term follow-up of a prospective, randomized, and multicenter study, we compare the results of a group receiving laparoscopic incisional ventral hernia repair using intraperitoneal onlay mesh (LG) to a group receiving a hybrid hernia repair where open closure of fascial defect was added to intraperitoneal mesh placement (HG).

**Methods:**

Originally, 193 patients with 2–7 cm incisional hernias were randomly assigned to either the LG or HG during the 30-month recruitment period in 2012 to 2015. Long-term follow-up was conducted 5–10 years after surgery to evaluate hernia recurrence rate and quality of life (QoL).

**Results:**

In all, 65 patients in the LG and 60 in the HG completed the long-term follow-up with a median follow-up period of 87 months. Recurrent hernia was detected in 11 of 65 patients (16.9%) in the LG and 10 of 60 patients (16.7%) in the HG (*p * >  0.9). Kaplan–Meier analysis demonstrated a recurrence rate approaching 20% in both groups, with similar curves. Three patients in the LG (4.6% and five patients in the HG (8.1%) had undergone re-operation due to recurrence (*p*  = 0.48). There was no difference in patient-reported QoL measured using the SF-36 questionnaire. Mean pain scores were similar between groups, mean numeric rating scale (NRS) 0 to 10 being 1.1 in the LG and 0.7 in the HG (*p*  = 0.43).

**Conclusion:**

Fascial closure did not reduce hernia recurrence rate in this study population, even though it has been shown to be beneficial and recommended in surgery guidelines. In the long term, recurrence rate for both groups is similar.

## Introduction

Incidence of incisional hernia after laparotomy is around 10% [1, 2]. Laparoscopic incisional ventral hernia repair (LIVHR) has gained in popularity over open repairs since it reduces postoperative surgical complications and complications-related re-operations [3]. In the original procedure for laparoscopic incisional hernia repair using intraperitoneal onlay mesh (IPOM), the fascial defect was left unclosed. This may lead to seroma formation or bulging [4]. In IPOM plus or a hybrid operation, a fascial defect is closed with sutures. Closing the fascial defect in a laparoscopic operation seems to improve patients’ quality of life (QoL) to a higher degree than merely bridging the defect [5]. In a recent meta-analysis, hybrid technique led to less surgical-site occurrences (SSOs) and less SSOs requiring intervention (SSOPI) compared to laparoscopic operations [6].

Our short-term results of the prospective, randomized, and controlled multicenter trial comparing hybrid and conventional LIVHR have been previously published. There were fewer and smaller seromas in the HG at one-month follow-up. No difference was found in hernia recurrence rates one year after operation and QoL was significantly improved from preoperative status in both groups [7, 8]. The aim of this long-term follow-up was to evaluate recurrence rate, QoL, pain, and re-operations due to any cause.

## Patients and methods

**Fig. 1:**
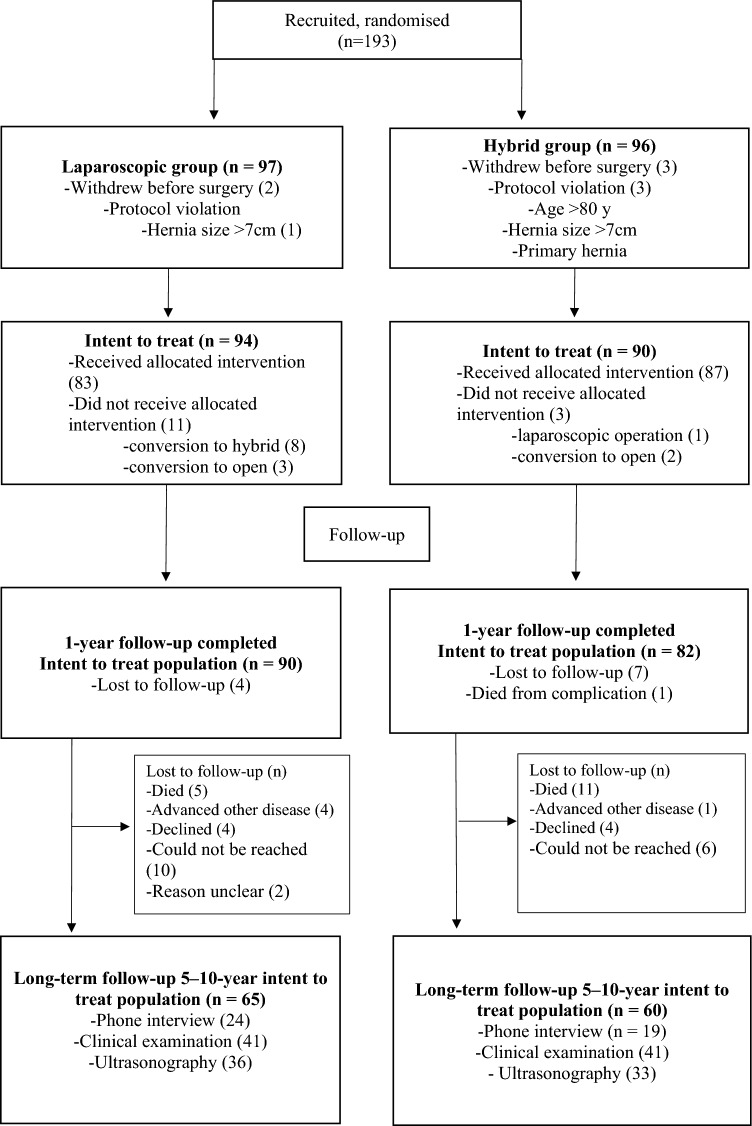
Patient flowchart

Eleven Hospitals participated in the study from November 2012 to May 2015, patients were randomized in blocks to have either a laparoscopic or hybrid operation for incisional ventral hernia repair. The trial was registered in Clinicaltrials.gov with the code NCT02542085 and the study design was discussed in detail in our research group previous publication of short-term results [7, 8]. In short, inclusion criteria were incisional hernia in patients aged 18–80. Exclusion criteria were American Society of Anesthesiologists score (ASA) > 4, body mass index (BMI) > 40, a previous mesh repair, width of hernia defect under 2 cm or over 7 cm, emergency operation, and the impossibility for adequate follow-up. Originally, 193 patients were randomly assigned to either the laparoscopic group (LG) or the hybrid group (HG) during the 30-month recruitment period seen in the flow chart (Fig. 1).

The primary outcome of this study is hernia recurrence rate. A patient was registered as having a recurrence if he or she was operated on for recurrence, or recurrence was detected in clinical or ultrasonographic examination or any other radiological examination. Secondary outcomes are QoL, re-operation due to any cause and pain at the hernia site.

Patients who had originally agreed to participate in the trial were invited for follow-up at the outpatient clinic at each attending hospital. They received information on the study and its significance, and written consent was obtained. The long-term follow-up was approved by the local ethical committee.

For the patients in the laparoscopic group (LG), hernia repair was performed as a standard IPOM procedure using a Parietex® composite mesh (Covidien), which was fixed to the peritoneum with the double crown technique using the Securestrap® tacking device (Ethicon) and four transabdominal sutures to anchor the mesh. In the hybrid group (HG), the hernia sack was resected and the fascial defect was closed with a slowly absorbing monofilament suture through a minilaparotomy incision before laparoscopic IPOM repair using the same mesh and fixation as in the LG.

Patients were invited to a clinical follow-up visit, which included clinical assessment by a surgeon and ultrasound examination of the hernia site. Long-term follow-up visits were arranged during year 2021. Ultrasound examination was performed at rest and also while using Valsalva´s maneuver. Recurrent hernia was defined as a fascial defect larger than 1 cm into which intra-abdominal fat or intestine could bulge. Pain score was registered with a numeric rating scale from 0 to 10 and QoL was measured using the SF-36 questionnaire [9].

Because of the COVID-19 pandemic, in one participating hospital, follow-up visits were not possible. Some patients also reported that because of the pandemic, they were unable or not willing to come to the follow-up visit. These patients were interviewed on the phone and re-operations and recurrences were confirmed from medical records.

## Statistics

**Fig. 2:**
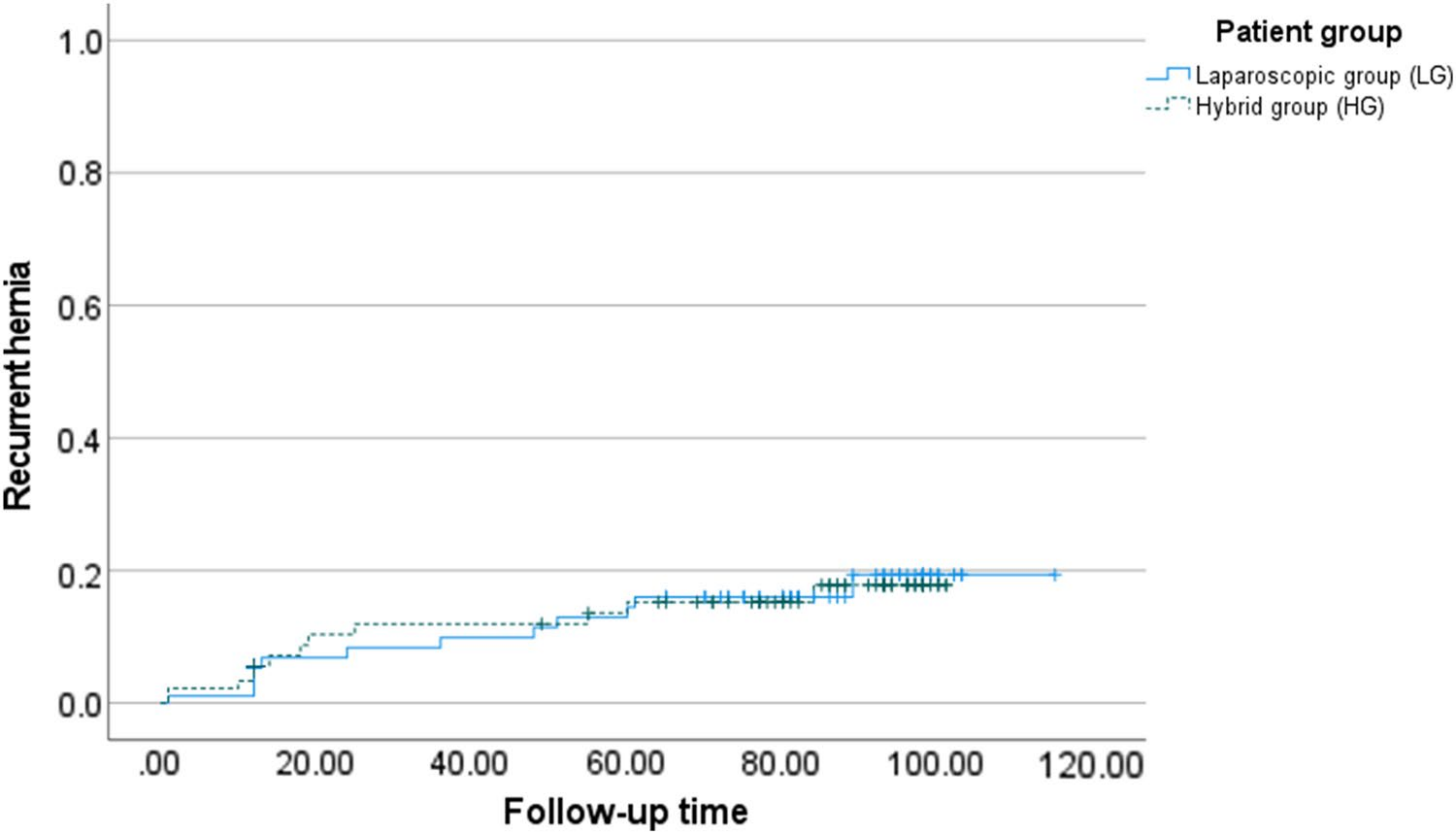
The Kaplan–Meier plot for hernia recurrence

Summary measurements are presented as means with standard deviations (SD), unless otherwise stated. A Kaplan–Meier curve was plotted for time to hernia recurrence for both groups and a log-rank test was used to compare groups. SPSS (IBM Corp. Released 2020. IBM SPSS Statistics for Windows, Version 27.0. Armonk, NY: IBM Corp) was used to create the Kaplan–Meier curve in Fig. 2. For continuous variables with normal distribution, independent-sample t-test was used to compare means. When a normal distribution assumption could not be made, the Mann–Whitney t-test was used to compare means. Categorical values were tested with Pearson Chi-Square when appropriate. Fisher´s exact test was used for categorical variables when > 25% of cells had an expected count of less than five. Unless otherwise stated, all results are analyzed and reported in intention to treat populations. Additional analyses for the primary outcome hernia recurrence were also conducted in per-protocol and as-treated patient populations. QoL data was collected also preoperatively, one month and one year postoperatively; therefore, we used Repeated Measures Mixed Model (RMMM) ANOVA to compare QoL for the long-term follow-up study. Time, group, and time*group were used as fixed effects and patient as a random effect in the RMMM. Two-tailed p-values are reported. Statistical programs SPSS (IBM Corp. Released 2020. IBM SPSS Statistics for Windows, Version 27.0. Armonk, NY: IBM Corp) and SAS (version 9.4, SAS Institute Inc., Cary, NC, USA) were used for analyses.

## Results

Long-term follow-up data were available in 65 patients in the LG and 60 patients in the HG with a mean follow-up time of 87 months for the LG and 87 months for the HG (*p* = 0.7). The resulting follow-up rate of the intent to treat population was 69.1% in the LG and 66.6% in the HG (*p* = 0.72). Reasons for losses to follow-up are listed for both groups in the flow chart (Fig. 1.). The most common cause for losses to follow-up was death or inability to participate because of weakened general condition. Of those patients not willing to come to the follow-up visit, 24 in the LG and 19 in the HG were interviewed on the phone.

In the LG 6% and in the HG 13% of patients died during the follow-up period (*p* = 0.097). Causes of death were not related to long-term complications of incisional hernia repair. Five patients had developed advanced disease, mostly dementia, which was an exclusion criterion for long-term follow-up. From both groups together, 8 patients declined and 16 patients could not be reached despite multiple efforts. The reason for lost to follow-up was unclear in 2 patients.

Baseline clinical characteristics are presented in Table 1. Groups were similar in terms of comorbidities. Mean hernia width was larger in the LG (4.2 cm vs 3.5 cm, *p* = 0.035), no difference was found in hernia height (3.7 cm vs 3.2 cm, *p* = 0.083).

**Table 1:** Baseline characteristics of patients completing long-term follow-up

	Laparoscopic group (LG) *n* = 65	Hybrid group (HG) *n* = 60	*p-*value
Age in years (at operation), mean (SD)	56.9 (11)	60.2 (12)	0.18
Female, *n* (%)	40 (61.5)	39 (65.0)	0.16
Cardiac disease, *n* (%)	30 (45.3)	27 (44.1)	0.49
Pulmonary disease, *n* (%)	10 (11.3)	10 (15.3)	0.80
Diabetes, *n* (%)	15 (19.4)	7 (10.2)	0.16
Smoking, *n* (%)	12 (18.5)	6 (10)	0.18
BMI, kg/m2 (SD)	31 (3.8)	30 (4.2)	0.52
Hernia width (cm), mean (SD)	4.2 (2.0)	3.5 (1.5)	0.035
Hernia height (cm), mean (SD)	3.7(1.6)	3.2 (1.4)	0.083
Mesh width (cm), mean (SD)	19.2 (3.9)	18.5 (4.7)	0.24

For continuous variables, standard deviation (*SD*) is presented

### Hernia recurrence

Recurrent hernia was detected in 11 of 65 patients (16.9%) in the LG and in 10 of 60 patients (16.7%) of the HG (*p* > 0.9). Recurrent hernia was diagnosed in clinical examination in 6.2% (*n* = 4) of patients in the LG and 3.3% (*n* = 2) of patients in the HG (*p* = 0.47). Ultrasonographic examination revealed or confirmed recurrence in 4 patients in the LG and 4 patients in the HG (*p* = 0.90). Results of the long-term follow-up are reported in Table 2.

**Table 2:** Results

	Laparoscopic group (LG) *n* = 65	Hybrid group (HG) *n* = 60	difference LG vs HG (95% CI)	*p-*value
Hernia recurrence, *n* (%)	11 (16.9%)	10 (16.7%)	0.25% (− 13–13)	> 0.9
Re-operation for recurrence, *n* (%)	3 (4.6%)	5 (8.3%)		0.48
Recurrent hernia in clinical examination, *n* (%)	4 (6.2%)	2 (3.3%)		0.47
Recurrent hernia in ultrasound, *n* (%)	4 (6.2%)	4 (6.6%)		0.90
Abdominal operation from any reason, *n* (%)	14 (21.5%)	12 (20%)	1.5% (-13–16)	0.80
Operation for bowel obstruction, *n* (%)	2 (3.1%)	0 (0%)	3.1% (-0.1–7.2)	0.50
Pain NRS, 0–10 (SD)	1.1 (2.3)	0.7 (1.8)	0.4 (-1.1–0.4)	0.43
Follow-up time (months), mean (range, SD)	87 (65–115, 12)	87 (65–101, 11)		0.77

For continuous variables, standard deviation (*SD*) is presented

95% Confidence interval (*CI*)

The Kaplan–Meier plot for hernia recurrence is shown in Fig. 2. Curves are almost identical, with recurrence rate approaching 20% 10 years after primary operation. Patients who dropped out during or before long-term follow-up are also included in the Kaplan–Meier plot, explaining the difference in number of recurrences compared to previous numbers. Three patients (4.6%) in LG and 5 patients (8.2%) in HG had undergone re-operation for recurrence (*p* = 0.48).

Per-protocol analysis with exclusion of patients who were converted during surgery to the other group showed an 18.3% (*n* = 11/60) recurrence rate in LG and 16.7% (*n* = 10/60) recurrence rate in HG (*p* = 0.81).

Additional as-treated analysis showed 16.9% (10/59) recurrence rate in patients who successfully underwent laparoscopic surgery and 14.1% (9/64) in patients who received a hybrid operation (*p* = 0.66).

Two patients in the LG had undergone operation for bowel obstruction and there were no re-operations in the HG for other reasons than recurrence (*n* = 2 vs. *n* = 0, *p* = 0.50).

At the time of long-term follow-up, QoL was similar between groups. Qol in eight concepts is summarized in Table 3. There was no difference in any of the eight scales. Patient-reported pain at the hernia site was low in both groups. Mean pain score (NRS from 0 to 10) was 1.1 (SD 2.3) in the LG and 0.7(SD 1.8) in the HG (p 0.43). Most patients reported pain to be 0 (*n* = 45 in LG and *n* = 43 in HG), with values ranging from 0 to 10 in the LG and 0 to 8 in the HG.

**Table 3:** Long-term SF-36 scores in laparoscopic and hybrid groups

	Laparoscopic group (LG) *n* = 47	Hybrid group (HG) *n* = 43	LG vs HG (95% CI)	*p*-value
Mental health, Mean (SD)	75 (22)	79 (19)	− 1.9 (− 9.0–5.2)	0.60
Physical role limitations, Mean (SD)	68 (40)	66 (40)	− -0.9 (− 17─15)	< 0.90
Bodily pain, Mean (SD)	69 (29)	68 (25)	1.1 (− 9.0–11)	0.83
General health, Mean (SD)	55 (23)	59 (21)	− 1.8 (− 9.1–5.6)	0.64
Vitality, Mean (SD)	62 (27)	68 (23)	− 3.2 (− 12–5.2)	0.46
Social functioning, Mean (SD)	75 (30)	83 (23)	− 5.7 (− 16–4.3)	0.26
Emotional role limitations, Mean (SD)	75 (39)	80(38)	− 7.0 (− 23–8.5)	0.38
Physical functioning, Mean (SD)	70 (30)	72 (29)	− 2.6 (− 13–7.4)	0.61

Mean values of scales and Standard deviation (*SD*)

## Discussion

Contrary to our hypothesis, open closure of facial defects did not reduce hernia recurrence. European Hernia Society (EHS) and American Hernia Society (AHS) guidelines recommend closure of fascial defects when feasible [10]. Defect closure is shown to reduce adverse hernia-site occurrences in retrospective reports and in our previous report [8, 11, 12]. Closing the defect is shown to reduce recurrences in non-randomized retrospective series [11]. To our knowledge, this is not proven in the RCT setting. The results of the recent RCT demonstrated improvement in QoL with defect closure, but no difference in hernia recurrence rate was found [5]. The optimal suture material for closing the defect is not clear. An absorbable suture was used in this study. In a later study, closing the defect with an absorbable suture did not reduce recurrences [13]. Modern ventral hernia surgery aims to restore abdominal midline and function of the abdominal wall. Even though our study did not show a decrease in hernia recurrence when fascial defect was closed, we do recommend defect closure when possible, in consideration of previously published evidence. Hernia repair should result in stable reconstruction, which can withstand increased abdominal wall strain. All aspects (mesh, sutures, and tackers) of repair should have adequate safety margins to support the forces caused by increased abdominal pressure for example when coughing. Closing the defect can sometimes create a stress concentration area, which ultimately leads to the failure of repair. The closure of fascial defect can increase or even decrease the durability of the repair and its outcome is dependent on the size and shape of the defect according to an experimental animal tissue model study [14]. The biomechanical durability of repair can be calculated or estimated and with computed tomography, repair can be individually planned to ensure adequate stability [15, 16]. At the time of the study, individual planning of the repair with the aim of ensuring adequate gained resistance toward impacts related to pressure was not in routine use.

Incidence of recurrences approaching 20% in Kaplan–Meier analysis and about 17% in patients completing long-term follow-up, with a median follow-up time of 87 moths, is in line with previously published studies. This demonstrates a recurrence rate of around 20%, and the fact that 20% of incisional hernia repairs are recurrent repairs supports this number [17]. Previously published recurrence rates have a large variance ranging from 0 to 61% [18].

One reason for the high long-term recurrence rate in this and previous studies might be the use of absorbable tackers for mesh fixation. Our results are in line with a previously published study where authors reported a recurrence rate of 28.5% at a median follow-up of 40 months when using absorbable mesh fixation and 18% when using nonabsorbable tacks [19]. Further, a Danish registry study concluded that closing the defect decreases re-operations for recurrence from 7.2% to 3.6% but only when securing the mesh with permanent tackers. They found no difference in re-operation rate when using absorbable tackers [20]. A Cochrane Review from 2021 did not find a difference in terms of recurrence between absorbable and nonabsorbable tacks. Although, the quality of evidence from randomized controlled trials (RCTs) is low with only 101 patients and a short follow-up time compared to that included in this analysis [21]. Less than half of the patients with recurrent hernia in this study underwent re-operation for recurrence, indicating that most of the recurrent hernias were asymptomatic or causing minor symptoms. Re-operation rates from recurrence (4.6% in LG and 8.2% in HG) were similar to the Danish registry [20].

According to the study protocol, mesh width had to be at least three times the fascial defect width or mesh overlap had to be at least 5 cm. Currently, guidelines recommend that the size of the mesh should be at least four times larger than the defect, or in other words, the mesh area-to-defect area ratio should be 16:1. The protocol allowed a smaller than currently recommended mesh size which may influence in recurrence rate, although on average, the mesh overlap is in line with recent guidelines.

There was a relatively large number of conversions to hybrid operation in the laparoscopic group (8/94). One patient in the HG received a laparoscopic operation. Even in per-protocol or as-treated analyses, the main result remained the same with no difference between groups in terms of recurrences, indicating this did not cause major inaccuracy.

One concern regarding intraperitoneal mesh placement is the possibility of mesh causing intraperitoneal adhesions which can lead to intestinal obstruction. Based on this study, intraperitoneal adhesions because of mesh rarely become a problem.

There are several limitations to this study that should be emphasized. According to power calculations, this study was originally designed to recruit 400 patients. However, we failed to reach this number, even with extension of the recruitment period. After the EHS and AHS recommendations for defect closure, surgeons became less active in recruiting patients. Thus, the number of surgeries per hospital remained small in some hospitals. Most patients were operated in four larger centers. However, all surgeons involved in the study were already experienced in laparoscopic incisional hernia surgery. We did not have a complete screening log of patients evaluated for the study. During the study years, tailoring the surgical technique and materials in accordance with modern biomechanical aspects was not in routine use. More individualized surgical techniques should be used to ensure that sufficient biomechanical durability has been achieved at the end of the surgery. The number of patients lost to follow-up was relatively high. Some of this was inevitable as in every study presenting long-term outcomes, and somewhat coincidental due to the COVID-19 pandemic. Adherence rate to the follow-up visit was similar in both groups. Efforts were made to keep adherence rate as high as possible and most patients were contacted multiple times and finally interviewed on the phone. However, despite these efforts, the high lost to follow-up rate may still represent a source of bias in this study.

## Data Availability

The data that support the findings of this study are available from the corresponding author upon reasonable request.

## Abbreviations

ASA: American Society of Anesthesiologists
BMI: Body mass index
HG: Hybrid group
NRS: Numeric rating scale
IPOM: Intraperitoneal onlay mesh
LG: Laparoscopic group
LIVHR: Laparoscopic incisional ventral hernia repair
SSO: Surgical-site occurrence
SSOPI: Surgical-site occurrence requiring intervention
QoL: Quality of life
